# Uric acid is associated with vascular dementia in Chinese population

**DOI:** 10.1002/brb3.617

**Published:** 2016-12-05

**Authors:** Yuzhen Xu, Qian Wang, Ruiting Cui, Kaili Lu, Yunlin Liu, Yuwu Zhao

**Affiliations:** ^1^ Department of NeurologyShanghai Jiao Tong University Affiliated Sixth People's HospitalShanghaiChina; ^2^ Department of Central LaboratoryTaishan Medical University Affiliated Taishan HospitalTaianShandong ProvinceChina; ^3^ Department of NeurologyTaishan Medical University Affiliated Taishan HospitalTaianShandong ProvinceChina

**Keywords:** oxidative stress, uric acid, vascular dementia

## Abstract

**Objective:**

Mounting evidence suggests that oxidative stress is involved in the pathogenesis of vascular dementia (VD). Uric acid (UA) has long been implicated as a critical cause of cardiovascular disease. Nevertheless, UA was also expected to play an important role in antioxidant and neuroprotection recently. We hypothesized that UA may have a protective role against VD. The aim of this study was to investigate the link between serum UA and cognitive dysfunction in VD.

**Materials and Methods:**

There were altogether 127 VD subjects and 81 nondemented controls enrolled in our study. Serum UA, demographic, and clinical characteristics were recorded at baseline, and all participants underwent Mini‐Mental State Examination (MMSE) at the beginning of the trial.

**Results:**

The VD group showed lower MMSE scores and serum UA levels than nondemented controls and there was significant statistical difference between the two groups (*p *< .05). Demographic and clinical characteristics such as age, gender, education, body mass index (BMI), total cholesterol (TC), triglycerides (TG), high‐density lipoprotein cholesterol (HDL), low‐density lipoprotein cholesterol (LDL), blood urea nitrogen (BUN), and serum creatinine (Scr) did not differ dramatically between groups (*p *> .05). In VD subjects, there was a positive correlation between serum UA and MMSE scores (*r* = .32, *p *<* *.05), and this correlation was independent of demographic and clinical characteristics (β = .272, *p *<* *.05).

**Conclusions:**

VD subjects have dramatically lower serum UA levels in comparison to nondemented controls. Lower serum UA levels are linked to cognitive dysfunction and could serve as a potential predictor for VD.

## 1 Introduction

As reported by the World Health Organization, approximately 35.6 million people are suffering from dementia and the figure is anticipated to have a twofold increase by 2030 and more than a threefold increase by 2050 (Foltyn, 2015).Vascular dementia (VD), one of leading dementia only second to Alzheimer's disease (AD), is accounting for no <15% of cases of dementia (O'Brien & Thomas, 2015). Each year there emerge an average of 7.7 million new dementia cases, which impose a heavy burden on families and social economy (Iadecola, 2013). However, current treatments are only modestly effective and there are no licensed treatments for VD. Hence, looking for potential hazard factors and exploring the prophylaxis and treatment are most important.

Uric acid (UA) is an end oxidative product of purine metabolism. UA has been known as a hazard factor of cardiovascular disease (Feig, Kang, & Johnson, 2008). In addition, high UA levels are also reported contributing to hypertension, the metabolic syndrome, chronic kidney disease, and diabetes, which are involved in various vascular diseases (Kumral, Karaman, Orman, & Kabaroglu, 2014; Molshatzki, Weinstein, Streifler, Goldbourt, & Tanne, 2015). The function of UA was mediated by an excessive increase in proliferation of vascular smooth muscle cell, an inflammatory response initiated by soluble UA and an impaired nitric oxide production, which directly damaged endothelial function (Simao, Lozovoy, & Dichi, 2012).

However, UA was also proposed to have diverse antioxidant effects such as the eliminating free radicals including hydroxyl radicals, hydrogen peroxide, and peroxynitrite, as well as the preventive effects against several kinds of oxidation reaction including the chelation of transition metals, the Fenton reaction, and lipid peroxidation (Amaro, Llull, & Renu, 2015). UA is thought to be the most important antioxidant and accounts for about 60% of the total antioxidant ability in humans. Over the past years, higher UA levels have been associated with slower progression of a variety of neurodegenerative disease including multiple sclerosis (MS), multiple system atrophy (MSA), Parkinson disease (PD), Huntington's Disease (HD), and AD, but limited data exist regarding VD (Kutzing & Firestein, 2008; Ndrepepa, Braun, & King, 2013).

To our knowledge, the pathogenesis of VD has not been completely elucidated, and it is yet unclear whether UA play a key role in the occurrence and development of VD. Mounting evidence suggests that oxidative stress is involved in the pathogenesis of VD (Liu & Zhang, 2012). Moreover, UA was also expected to play an important role in antioxidant and neuroprotection by reducing oxidative stress and protecting against free radicals (de Giorgi, Fabbian, & Pala, 2015). We hypothesized that UA may have a protective role against cognitive dysfunction in VD. If this assumption is confirmed it would be of great clinical and public health importance.

## 2 Materials and Methods

### 2.1 Participants

From January 2015 to March 2016, 127 VD patients admitted to Department of Neurology in Taishan Medical University Affiliated Taishan Hospital, Taian, Shandong Province, China, were consecutively and prospectively enrolled in the study. According to the (DSM‐IV) (Association A, 2000) and National Institute for Neurological Disorders and Stroke (NINDS‐AIREN) (Roman, Tatemichi, & Erkinjuntti, 1993), VD was diagnosed by neurological physicians. In parallel, 81 controls who were functionally independent and cognitively healthy were randomly chosen from different departments of the same hospital. The controls were selected in such a way that the following criteria matched the patients: eating habits, age, and BMI. The control group and the patient group have similar educational levels. Subjects who had a known history of severe mental disorders, hypertension, diabetes, renal disease, tumor, antihyperuricemic drugs, and substance abuse were excluded. Demographic characteristics were recorded including age, gender, education, and body mass index. The study was authorized by the Human Ethics Committees of Taishan Medical University Affiliated Taishan Hospital and we obtained informed consent from all study participants in accordance with the Helsinki Declaration.

### 2.2 Cognitive function testing

The Mini‐Mental State Examination (MMSE) is a most widely used scale for screening and assessing cognitive decline, which acts as one of the essential tests recommended according to the NINDS‐AIREN. The total score of MMSE is 30 points. MMSE comprises seven sections including Time orientation (5 points), Space orientation (5 points), Attention and Calculation (5 points), Retell of three words (3 points), Registration of three words (3 points), Linguistic Function (8 points), and Visual Performance (1 point) (Cui, Yao, & Xu, 2011). A score of <24 generally is generally regarded as one of the important signs of significant cognitive dysfunction (Li, Jia, & Yang, 2016; Tombaugh & McIntyre, 1992). The test was performed at standard conditions with an average of 45 minutes. The time to administer MMSE test and score was recorded. The attending physicians were blinded to all the variables of participants.

### 2.3 Uric acid and other clinical characteristics measurement

All fasting for at least 8 h and after that peripheral venous blood samples were collected in the morning from all participants who were still in a recumbent position (Zhu, Zou, Xiong, & Zhang, 2016). Venous blood (5 mL) was drawn into an EDTA containing tube and then within 30 min the sample was centrifuged at 3000 rpm for 15 min to obtain the serum, which was then stored at −80 °C refrigerator (Tao, Hu, & Wu, 2012). Serum UA (SUA), total cholesterol (TC), triglycerides (TG), high‐density lipoprotein cholesterol (HDL), low‐density lipoprotein cholesterol (LDL), blood urea nitrogen (BUN), and serum creatinine (Scr) were determined with commercial kits and an automatic blood cell analyzer (Hitachi 747; Hitachi, Tokyo, Japan) at the central laboratory of Taishan Medical University Affiliated Taishan Hospital.

### 2.4 Statistical analysis

The SPSS statistical package for windows version 20.0 (SPSS Inc., Chicago, IL, USA) was used for data analysis. Continuous variables (MMSE scores, age, education, BMI, UA, TC, TG, HDL, LDL, BUN and Scr) were summarized as means ± SD and categorical variables (gender) were summarized as frequency and percentages. Differences between VD patients and nondemented controls were examined using the Student's *t*‐tests and the χ^2^ test for continuous variables and categorical variables, respectively. For the correlation of MMSE score with serum UA, age, gender, education, BMI, UA, TC, TG, HDL, LDL, BUN, and Scr the Pearson correlation coefficient was used. Multiple linear regression was applied to evaluate the forecast value of different variables on MMSE scores. A two‐tailed *p *<* *.05 was regard as dramatically different.

## 3 Results

From January 2015 to March 2016, 127 VD patients [mean age ± SD: 67.4 ± 7.8, 69 males (54.3%)] and 81 controls [mean age ± SD: 68.1 ± 8.2, 43 males (53.1%)] with normal cognitive function were evaluated. A comparison of demographics and clinical characteristics at baseline are summarized in Table 1. Mean values of serum UA and MMSE scores of VD patients were dramatically lower than nondemented subjects (*p *=* *.018 vs. *p *<* *.001, respectively). Years of education of VD patients were lower than nondementia controls, though there was no distinct difference between them (*p *>* *.05). The characteristics regarding age, gender, years of education, BMI, TC, TG, HDL, LDL, BUN, and Scr were not markedly different between groups (*p *>* *.05).

**Table 1:** Baseline characteristics of subjects

Characteristics	Controls (*n *= 81)	VD patients (*n *= 127)	*p*
Age, years	68.1 ± 8.2	67.4 ± 7.8	.537
Male, n (%)	43(53.1%)	69(54.3%)	.861
Education, years	8.5 ± 2.4	8.3 ± 3.3	.638
BMI, Kg/m^2^	24.6 ± 1.8	24.9 ± 1.5	.195
TC, mmol/L	4.33 ± 0.60	4.43 ± 0.56	.223
TG, mmol/L	1.53 ± 0.21	1.55 ± 0.27	.572
HDL, mmol/L	1.30 ± 0.18	1.27 ± 0.20	.274
LDL, mmol/L	2.62 ± 0.31	2.69 ± 0.43	.206
BUN, mmol/L	4.95 ± 1.16	5.13 ± 1.13	.269
Scr, μmol/L	66.43 ± 8.82	67.76 ± 11.17	.366
SUA, μmol/L	336.59 ± 103.63	300.12 ± 110.48	.018
MMSE	26.5 ± 1.2	20.3 ± 1.7	<.001

VD, vascular dementia; BMI, body mass index; TC, total cholesterol; TG, triglycerides; HDL‐C, high‐density lipoprotein cholesterol; LDL‐C, low‐density lipoprotein cholesterol; BUN, blood urea nitrogen; Scr, serum creatinine; SUA, serum uric acid; MMSE, Mini‐Mental State Examination.

Pearson correlation analysis between MMSE scores and baseline data in VD patients are presented in Table 2. Pearson correlation analysis showed a significant negative correlation between MMSE scores and age (*r* = −.36, *p *=* *.034) and a significant positive correlation between MMSE scores and serum UA levels (*r* = .32, *p *=* *.022). Nevertheless, no statistically significant correlation between MMSE scores and other baseline characters including gender, years of education, BMI, TC, TG, HDL, LDL, BUN, and Scr was found (*p *>* *.05).

**Table 2:** Pearson correlation analysis between MMSE and variables of VD patients (*n *= 127)

	MMSE	Age	Gender	Education	BMI	TC	TG	HDL	LDL	BUN	Scr	SUA
MMSE												
Pearson Correlation	1.000	−0.36	0.08	0.282	−0.091	−0.095	0.002	0.085	−0.041	−0.21	−0.167	0.32
Sig. (two‐tailed)		0.034	0.369	0.137	0.31	0.286	0.982	0.341	0.65	0.814	0.451	0.022
N	127	127	127	127	127	127	127	127	127	127	127	127

VD, vascular dementia; BMI, body mass index; TC, total cholesterol; TG, triglycerides; HDL‐C, high‐density lipoprotein cholesterol; LDL‐C, low‐density lipoprotein cholesterol; BUN, blood urea nitrogen; Scr, serum creatinine; SUA, serum uric acid; MMSE, Mini‐Mental State Examination.

The results of multiple linear regression analysis between MMSE scores and baseline data in VD patients are presented in Table 3. Multiple linear regression analysis showed an inverse association between MMSE scores and age in VD patients (β = −.23, *p *=* *.011). And multiple linear regression analysis also showed that there was a positive correlation between MMSE scores and serum UA levels of VD subjects (β = .272, *p *=* *.036). However, no statistically significance between MMSE scores and other baseline characters including gender, years of education, BMI, TC, TG, HDL, LDL, BUN, and Scr was found (*p *>* *.05).

**Table 3:** Linear regression between MMSE and variables of VD patients (*n *= 127)

Model	Standardized coefficients		95% CI for B	
	β	*p*	Lower bound	Upper bound
Age	−.23	.011	−0.47	0.17
Gender	−.109	.248	−0.268	0.028
Education	.108	.266	−0.154	0.43
BMI	−.081	.388	−0.107	0.12
TC	−.075	.427	−0.209	0.145
TG	.021	.82	−0.05	0.103
HDL	.114	.234	−0.137	0.231
LDL	−.098	.312	−0.169	0.176
BUN	−.103	.276	−0.298	0.129
Scr	.097	.307	−0.014	0.245
SUA	.272	.036	0.078	0.433

MMSE, Mini‐Mental State Examination; VD, vascular dementia; 95% CI, 95% confidence interval; BMI, body mass index; TC, total cholesterol; TG, triglycerides; HDL‐C, high‐density lipoprotein cholesterol; LDL‐C, low‐density lipoprotein cholesterol; BUN, blood urea nitrogen; Scr, serum creatinine; SUA, serum uric acid.

## 4 Discussion

In our study, the differences in serum UA levels and cognitive function between VD patients and nondemented controls were examined. The result suggested that VD patients have significant lower MMSE scores, in addition with reduced serum UA levels. Our study indicated that reduced serum UA levels might contribute to the pathogenesis of VD and there existed a possible link between VD and serum UA levels. We further evaluated the correlation between serum UA levels and MMSE scores. The results showed that there existed a significant positive correlation between MMSE scores and serum UA levels in patients with VD and the association was significant even after adjusting for the confounding factors. As far as we know, there are limited published papers investigating the correlation between serum UA levels and VD until now.

Currently we still have poor knowledge about the pathogenesis of VD, however, there exist powerful evidence provided by numerous studies that oxidative stress is involved in the development of VD (Liu & Zhang, 2012). Oxidative stress is a status that the amount of pro‐oxidant species exceeds the amount of antioxidant species and the balance between them is broken (Bennett, Grant, & Aldred, 2009; Xi, Yu, & Ding, 2012). Oxidative stress plays an important role in the occurrence and development of VD, which has been widely reported. Oxidative stress has been proposed as a hazard factor of VD (Luca, Luca, & Calandra, 2015). Y. Ihara and colleagues demonstrated that VD subjects had elevated levels of hydroxyl radical and reduced levels of superoxide dismutase in plasma compared to nondemented controls (Ihara, Hayabara, & Sasaki, 1997). Another study reported that the amount of oxidative DNA damage repair products excreted through the urethra and 8‐oxoguanine in cerebrospinal fluid is higher in mixed Alzheimer disease/vascular dementia than in the age‐matched nondemented controls, whereas the serum levels of ascorbic acid and retinol tend to be reduced (Gackowski, Rozalski, & Siomek, 2008). Recently a case–control study demonstrated that VD patients had significantly lower 8‐isoprostaglandin F2a (8‐isoPGF2a) and higher urinary 8‐hydroxydeoxyguanosine (8‐OHdG) levels in compared to controls (Shi, Liu, Wang, Guan, & Li, 2012). Taken together, all above results indicated that oxidative stress contributed to the occurrence and development of VD.

These findings support the theory that oxidative stress contribute to VD. It is less clear whether UA, an end oxidative product of purine metabolism accounting for 60% of the total antioxidant capacity in human plasma (Morimoto, Simao, & de Almeida, 2014), is important to the development or progression of VD. However, several studies have confirmed the correlation between UA levels and cognitive disorder. In a large prospective population‐based cohort study, S. M. Euser et al. suggested that higher levels of UA are associated with a decreased risk of dementia and better cognitive function (Euser, Hofman, Westendorp, & Breteler, 2009). In a prospective Alzheimer's disease (AD) study, B. S. Ye and colleagues found that elevated serum UA levels had neuroprotective effects, which could alleviate the longitudinal cognitive decline and serve as a valid indicator of cognitive decline independent of classical AD biomarkers (Ye et al., 2016). Recently, another study revealed that in a Mexican population higher serum UA levels are linked with a lower risk of cognitive decline (Mendez‐Hernandez, Salas‐Pacheco, & Ruano‐Calderon, 2015). In addition, it is also reported that people suffering from gout had a higher UA levels eventually putting them at a decreased risk of developing dementia (Hong et al., 2015; Lu, Dubreuil, & Zhang, 2016), which further indicated that UA has potential neuroprotective effect against dementia. Our results are consistent with all above mentioned studies suggesting that lower UA level tend to be a hazard factor for various cognitive dysfunction, however data regarding VD are limited.

UA is considered to be one of most abundant antioxidant existing in the blood. Nevertheless, UA can serve likewise as pro‐oxidant particularly in the condition exposed to high levels of singlet oxygen and peroxynitrite (Bowman, Shannon, Frei, Kaye, & Quinn, 2010). Thus, the role of UA in cognitive dysfunction is controversial. Two Italy studies showed that patients with AD and VD both had higher UA levels compared to health controls (Cervellati et al., 2013, 2014). Similarly, another study found that high plasma UA levels contributes to an increased risk of developing dementia in elderly Italian (Ruggiero, Cherubini, & Lauretani, 2009). Moreover, it is also reported that higher baseline serum UA is related to cognitive decline in women (Vannorsdall, Kueider, Carlson, & Schretlen, 2014). In contrast, our study indicated that VD subjects had dramatically lower plasma UA concentrations compared to controls. Three above mentioned studies were conducted in Italian and one studies was conducted only in women, the differences in race, gender ratio, and dementia types might partly explain the conflicting outcomes.

In our study, the results suggested that there was an obvious positive correlation between MMSE scores and serum UA levels and that lower serum UA levels was an independent risk factor in VD. As far as we know, there are no published papers investigating the correlation between UA levels and MMSE scores in VD until now. Besides UA levels, age is another independent risk factor for VD (Erkinjuntti, Laaksonen, Sulkava, Syrjalainen, & Palo, 1986; Sahathevan, Brodtmann, & Donnan, 2012). Our study showed that age was inversely correlated with MMSE scores and the association was independent of confounding factors, which were consistent with previous studies (Al‐Khateeb, Althaher, & Al‐Khateeb, 2015). The incidence of dementia increases with age. With aging, cerebral blood flow (CBF) gradually decreases, which are partly involved in the pathogenesis of VD (Raz, Knoefel, & Bhaskar, 2016). Moreover, the number of elderly is expected to increase exponentially in the next few decades (Christensen, Doblhammer, Rau, & Vaupel, 2009). However, aging, the most important reason for dementia, is beyond any such measures at present. Thus, searching for any modifiable risk factors such as UA is more important.

Our study has some limitations. First, there were relatively few participants enrolled in our research. Second, the VD groups may comprise subjects with mixed dementia. Third, all the participants are Chinese, so whether the results in our study are appropriate for other ethnic groups is uncertain, which needs further research. Fourth, data on personal lifestyle such as the course and drug therapy history of VD, smoking status, and alcohol drinking status are not available. However, the merit of this study is that it is the first study on the relation between UA and VD in Chinese population.

Taken together, our major finding of this study was that lower serum UA level is strongly associated with cognitive dysfunction and serum UA level plays a protective role against cognitive decline in VD. Nevertheless, additional prospective large clinical trials are needed to evaluate whether appropriately increasing UA level could decrease the incidences of VD. The relationship between UA and cognitive dysfunction in VD, if proven by some larger clinical trials, may have important clinical and therapeutic promise.

## Conflicts of Interest

The authors declare no conflicts of interest in this study.
